# Construction and validation of a web-based dynamic predictive model for the risk of postoperative nausea and vomiting in patients undergoing day-case hysteroscopic surgery

**DOI:** 10.3389/fmed.2025.1582546

**Published:** 2025-07-23

**Authors:** Jiang Liu, Lifang Han, Fengxian Zhang, Yan Jiang, Lin Cheng, Sifan Qin, Shirong Fang

**Affiliations:** ^1^Xiangya School of Nursing, Central South University, Changsha, China; ^2^Department of Human Resources, Second People’s Hospital of Weifang, Weifang, China; ^3^Xiangya School of Nursing, Shandong Second Medical University, Weifang, China; ^4^Department of Anesthesiology, Weifang People’s Hospital, Weifang, China

**Keywords:** postoperative nausea and vomiting, PONV, day-case hysteroscopic surgery, predictive model, risk score

## Abstract

**Objectives:**

This study aimed to develop and validate a predictive risk model for postoperative nausea and vomiting (PONV) in patients undergoing day-case hysteroscopic surgery.

**Methods:**

The candidate predictors were identified by systematic literature review. Patients who met the study criteria were divided into training group and validation group. The time-period validation was used for the external validation of the model. The candidate predictors with statistical significance through lasso regression analyses were included in multifactor logistic regression analyses. The calibration and receiver operating characteristic (ROC) curves were utilized to assess the accuracy of model. Decision curve analysis (DCA) was used to assess the clinical benefit of Nomogram. All statistical analyses were constructed by RStudio software (version 4.2.1).

**Results:**

A total of five predictors were included in the PONV risk prediction model: (1) motion sickness (OR, 8.53; 95% CI, 6.21–11.81), (2) anesthesia time (OR, 4.20; 95% CI, 2.09–8.65), (3) fasting time (OR, 1.17; 95% CI, 1.13–1.22), (4) anxiety score (OR, 1.10; 95% CI, 1.08–1.12), and (5) artificial airway (OR, 0.54; 95% CI, 0.39–0.74). The area under the ROC curve for the training cohort and validation cohort was 85.0% (95% CI: 82.6–87.5%) and 80.3% (76.2–84.3%), respectively.

**Conclusion:**

The predictive model demonstrated potential in predicting the risk of PONV in patients undergoing day-case hysteroscopic surgery.

## Highlights

- Notably, the incidence of PONV in patients undergoing hysteroscopic day-case surgery was 36.0%.
- The nurses are in a key position to recognize and prevent PONV in patients undergoing hysteroscopic day-case surgery.
- A web-based dynamic prediction model constructed and validated is potentially useful to clinical nurses for early prediction and control of PONV risk in patients undergoing hysteroscopic day-case surgery.

## Introduction

Postoperative nausea and vomiting (PONV) is the most common postoperative adverse reaction among patients undergoing gynecological surgery (1–3), with an incidence 6.27 times higher than that of other types of surgery (4). With the growing acceptance of the enhanced recovery after surgery (ERAS) concept, ambulatory surgery has become increasingly prevalent. Approximately 25% of ambulatory surgical patients experience PONV either immediately after surgery or after discharge from the hospital (5). PONV can lead to delayed anesthesia recovery, unplanned hospital readmission and increased healthcare costs (2). Therefore, preventing and managing PONV in ambulatory surgery demands the same level of attention as postoperative pain management.

Hysteroscopic day-case surgery, as a common gynecological ambulatory surgery, has gained increasing acceptance among clinicians and patients due to its efficiency and safety (6, 7). Beyond the inherent risks of general anesthesia, cerebral edema and elevated PONV risk may result from acute hyponatremia and amino acid imbalances caused by excessive irrigation fluid absorption (8–10). Previous study indicated that patients remain vulnerable to PONV even after discharge from ambulatory surgery (11). Patients consistently report PONV as a highly undesirable complication (12), and those undergoing hysteroscopic procedures face greater recovery challenges. These include difficulties in accessing readmission and staff lack of preparation compared to other surgery. However, accurate prediction and effective management of PONV remain clinically challenging (13, 14).

Previous studies have consistently identified female sex as a common risk factor for PONV (15–17). However, there is ongoing debate regarding the many risk factors that may lead to PONV. Tan et al. reported that these factors may vary across different patient populations (18). Given the non-negligible side effects of antiemetics and patients’ preferences, prophylactic administration to all surgical patients is impractical. Furthermore, the reported incidence of PONV ranges from 9 to 56% (1, 19, 20). These findings highlight the importance of assessing individual risk factors to guide treatment and prevention strategies. A reliable predictive model may support clinical decision-making by facilitating patient recovery, reducing anxiety, and developing antiemetic strategies.

Therefore, the purpose of this study was to construct and validate a risk prediction model for PONV in patients undergoing hysteroscopic day-case surgery.

## Methods

### Study design and ethical approval

This is a prospective cohort study designed for patients undergoing day-case hysteroscopic surgery. The study was approved by the Institutional Review Board (IRB) of Weifang People’s Hospital (IRB KYLL20230217-3). The model was externally validated through the time-period validation method. Participants from January 2021 to January 2023 were consecutively collected for the modeling group, and from January 2023 to January 2024 for the external validation group. Written informed consent was obtained from all patients before the study began, and the study protocol was enrolled in the clinical trial registry (NCT06524752).

### Participant

Inclusion criteria: (1) patients (aged ≥ 18 years) undergoing hysteroscopic day-case surgery (including uterine polypectomy, submucous myomectomy, etc.), (2) informed consent provided and voluntarily participated in the study, (3) stable postoperative physiological status with no serious complications. Exclusion criteria: (1) patients with communication difficulties, (2) intraoperative perforation of the uterine cavity or massive bleeding required laparotomy, (3) persistent or recurrent nausea/vomiting prior to anesthesia, (4) administration of antiemetics such as ondansetron within 24 h before surgery, (5) surgery resulting in planned or unplanned hospitalization.

### Sample size

The sample size required for the multifactorial logistic regression analysis in this study was calculated using the sample size package in R software. The ratio of sample size between the modeling group and the validation group was 7:3. This ratio is currently recognized as optimal for providing stable results in risk prediction model construction. Considering a 30% expected incidence of PONV, a 20% loss-to-follow-up rate, and an R-squared of 0.1, a minimum of 1,457 participants were required for this study (training cohort = 1,020, validation cohort = 437).

### Anesthesia protocol

All patients inhaled pure oxygen for 2 min before induction of anesthesia. Intravenous dexamethasone (10 mg) was administered to prevent PONV at the commencement of surgery. Propofol (1.5–2 mg/kg), fentanyl (1–2 ug/kg), and rocuronium bromide (0.8 mg/kg) were utilized for induction and maintenance of anesthesia. Endotracheal intubation or laryngeal mask was utilized for construction of an artificial airway once the eyelid reflexes had disappeared, the muscles were relaxed, and conditions for intubation were met. Depth of anesthesia was monitored using Bispectral Index (BIS). Intraoperative blood pressure and oxygen levels were continuously monitored. All patients were infused intravenously with Ringer’s lactate solution (500 mL/h) as intraoperative maintenance fluid. The patient-controlled analgesia (PCA) pump was utilized for postoperative analgesia (nalbuphine in 50 mL saline, background infusion 2 mL/h for 24 h, bolus 2 mL, lockout time 15 min).

### Variables and measurements

The incidence of PONV (yes/no) was the dependent variable, and the candidate predictors related to PONV were the independent variables. Candidate predictors associated with PONV were included through a literature review and group discussion. Eventually, 18 candidate predictors were included in the lasso regression analysis. The details of the predictors are shown in Table 1. The results of lasso regression analyses are detailed in Table 2. The outcome of study was incidence of PONV. PONV was defined as epigastric discomfort, nausea and retching with or without vomiting that occurred within 24 h after surgery (21, 22). At 24 h postoperatively, the investigators assessed the incidence of nausea and vomiting in the patients through telephone follow-up. The motion sickness was recorded using the Visual Analog Scale (VAS) (23). The level of preoperative anxiety was evaluated using the Beck Anxiety Inventory (BAI) (24). The process of data collection was conducted by two independent investigators who were professionally trained and blinded to the purpose of this study. Other candidate risk factors, such as anesthesia time and the type of artificial airway were also collected via the electronic medical record system. The one-way analysis and LASSO regression were used to exclude possible confounding variables. Furthermore, a research assistant visited the patient preoperatively and recorded basic information, including age, weight, fasting time, length of surgery, and ASA classification.

**Table 1:** Characteristics and candidate risk factors of patients.

Characteristics	PONV (*n* = 570)	No PONV (*n* = 1,013)	*P*-value	Test group (*n* = 1,091)	Validation group (*n* = 492)	*P*-value
Age, (mean ± SD) (yr)	42.27 ± 9.32	42.57 ± 9.51	0.549	42.41 ± 9.42	42.57 ± 9.50	0.753
BMI, (mean ± SD)	23.98 ± 3.62	24.03 ± 3.53	0.819	24.02 ± 3.53	23.99 ± 3.64	0.904
ETco2, (mean ± SD)	40.83 ± 4.99	40.60 ± 4.90	0.369	40.80 ± 4.97	40.43 ± 4.83	0.162
ASA class, *n* (%)			0.148			0.89
I	69 (12.1)	99 (9.8)		115 (10.5)	53 (10.8)	
II	501 (87.9)	914 (90.2)		976 (89.5)	439 (89.2)	
Motion sickness, *n* (%)			<0.001			0.007
Yes	407 (71.4)	276 (27.2)		446 (40.9)	237 (48.2)	
No	163 (28.6)	737 (72.8)		645 (59.1)	255 (51.8)	
Anesthesia time, (mean ± SD) (hr)	0.64 ± 0.28	0.56 ± 0.22	<0.001	0.59 ± 0.24	0.60 ± 0.26	0.318
Artificial airway, *n* (%)			0.005			0.844
Laryngeal mask	290 (50.9)	589 (58.1)		604 (55.4)	275 (55.9)	
Trachea cannula	280 (49.1)	424 (41.9)		487 (44.6)	217 (44.1)	
BAI score, (mean ± SD)	40.35 ± 10.34	34.35 ± 10.13	<0.001	36.31 ± 10.55	36.96 ± 10.70	0.259
Type of operation, *n* (%)			0.202			0.497
Uterine polypectomy	430 (75.4)	723 (71.4)		803 (73.6)	350 (71.1)	
Submucous myomectomy	132 (23.2)	276 (27.2)		272 (24.9)	136 (27.6)	
Other	8 (1.4)	14 (1.4)		16 (1.5)	6 (1.2)	
Smoking, *n* (%)			0.846			0.426
Yes	4 (0.7)	8 (0.8)		7 (0.6)	5 (1.0)	
No	566 (99.3)	1005 (99.2)		1084 (99.4)	487 (99.0)	
Fasting time, (mean ± SD) (hr)	18.12 ± 4.63	16.21 ± 3.90	<0.001	16.94 ± 4.23	16.80 ± 4.36	0.56
Pain score, (mean ± SD)	4.82 ± 3.04	4.74 ± 3.06	0.625	4.74 ± 3.05	4.82 ± 3.06	0.652
Migraine, *n* (%)			0.624			0.414
Yes	192 (33.7)	329 (32.5)		352 (32.3)	169 (34.3)	
No	378 (66.3)	684 (67.5)		739 (67.7)	323 (65.7)	
Dexamethasone, *n* (%)			0.364			0.603
Yes	265 (46.5)	495 (48.9)		519 (47.6)	241 (49.0)	
No	305 (53.5)	518 (51.1)		572 (52.4)	251 (51.0)	
PCA, *n* (%)			0.503			0.272
Yes	109 (19.1)	180 (17.8)		207 (19.0)	82 (16.7)	
No	461 (80.9)	833 (82.2)		884 (81.0)	410 (83.3)	
Dexmedetomidine, *n* (%)			0.685			0.653
Yes	250 (43.9)	455 (44.9)		490 (44.9)	215 (43.7)	
No	320 (56.1)	558 (55.1)		601 (55.1)	277 (56.3)	
Sevoflurane, *n* (%)			0.317			0.517
Yes	63 (11.1)	96 (9.5)		106 (9.7)	53 (10.8)	
No	507 (88.9)	917 (90.5)		985 (90.3)	439 (89.2)	
Dezocine or Eptazocine, *n* (%)			0.002			0.563
Yes	437 (76.7)	841 (83.0)		885 (81.1)	393 (79.9)	
No	133 (23.3)	172 (17.0)		206 (18.9)	99 (20.1)	

PONV, postoperative nausea and vomiting; ASA, American Society of Anesthesiologists; BMI, body mass index; BAI, beck anxiety inventory; ETco2, end-tidal carbon dioxide; PCA, patient-controlled analgesia.

Partial references for risk factor pool construction: Apfel et al. (38), Stamer et al. (39), and Van den Bosch et al. (40).

**Table 2:** β-coefficients and Lambda. Min value of lasso regression.

Candidate predictor	β-coefficients	Lambda. Min
Age	−0.005429153	0.000583
BMI	−0.008433630	
ETco2	−0.005707187	
Pain score	−0.009644633	
Fasting time	0.164029983	
Anesthesia time	1.389262330	
BAI	0.094122407	
Type of operation	−0.141658745	
Artificial airway	−0.644576649	
Motion sickness	2.145775880	
Migraine	−0.121262572	
ASA	0.245536316	
Smoking	−0.375282641	
Dexamethasone	−0.202191733	
PCA	−0.075233741	
Dexmedetomidine	−0.198032043	
Sevoflurane	−0.302819132	
Dezocine or Eptazocine	0.010680179	

BMI, body mass index; ETco2, end-tidal carbon dioxide; BAI, beck anxiety inventory; ASA, American Society of Anesthesiologists; PCA, patient-controlled analgesia.

### Data analysis

Descriptive statistics, such as means and standard deviations (SD), were used to describe the continuous variables, and categorical variables were expressed as percentages. T-tests, one-way analysis of variance (ANOVA), and chi-square analysis were performed to determine the intergroup differences. Participants enrolled in this study were divided into modeling group and validation group. The methods and requirements of the model building group are consistent with those of the external validation group. To avoid model overfitting caused by screening numerous independent variables, candidate predictors were initially screened using lasso regression analysis. Statistically significant predictors by lasso regression were included in multifactor logistic regression analyses with stepwise selection. The results were presented as odds ratio (OR) with 95% confidence intervals (CI). The accuracy of model was evaluated by generating receiver operating characteristic (ROC) curves and calculating the areas under the curves (AUC). Hosmer-Lemeshow (HL) test was performed to assess the goodness of fit of the model. Decision curve analysis (DCA) was used to assess the clinical benefit of Nomogram. Finally, calibration curve was used to evaluate the goodness-of-fit of the model. All statistical analyses were performed by a statistician blinded to the purpose of the study using RStudio software (version 4.2.1).

## Results

**Figure 1:**
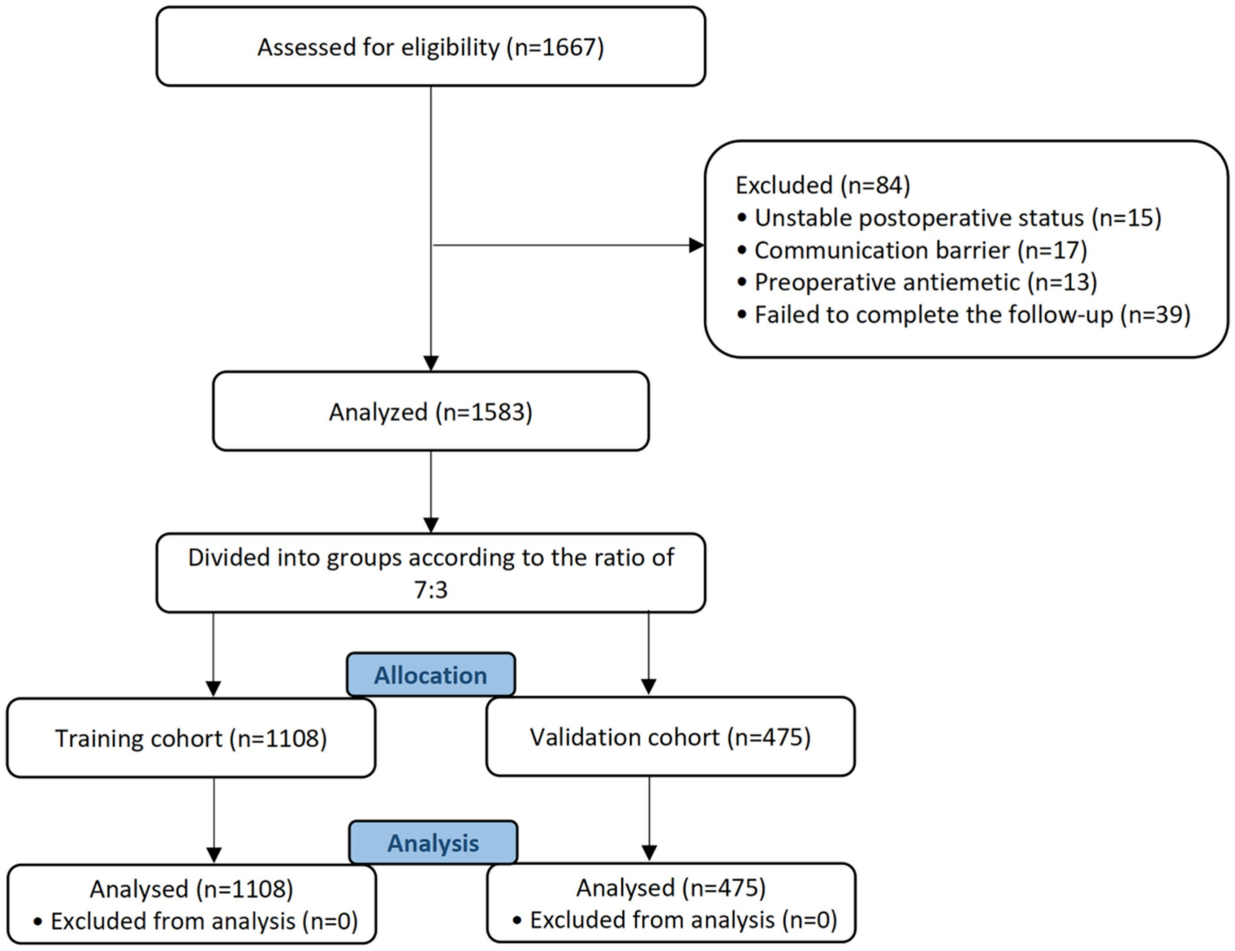
Flow chart for patient registration.

A total of 1,667 individuals were recruited for this study (Figure 1). Eighty-four patients were ruled out of this study due to unstable postoperative status (*n* = 15), communication barriers (*n* = 17), use of preoperative antiemetics (*n* = 13), and failure to complete follow-up (*n* = 39).

### Characteristics of patients

There are 36.0% participants experienced PONV. The average age and BMI of the participants were 42.5 ± 9.4 years and 24.0 ± 3.6, respectively. The average anesthesia time for participants was 0.59 ± 0.25 h, and the ASA classification was 1 to 2. For the type of intraoperative artificial airway (endotracheal intubation or laryngeal mask), 879 participants received a laryngeal mask and 704 received a endotracheal intubation of all participants. The characteristics of the patients are detailed in Table 1.

### Candidate predictors associated with PONV

**Figure 2:**
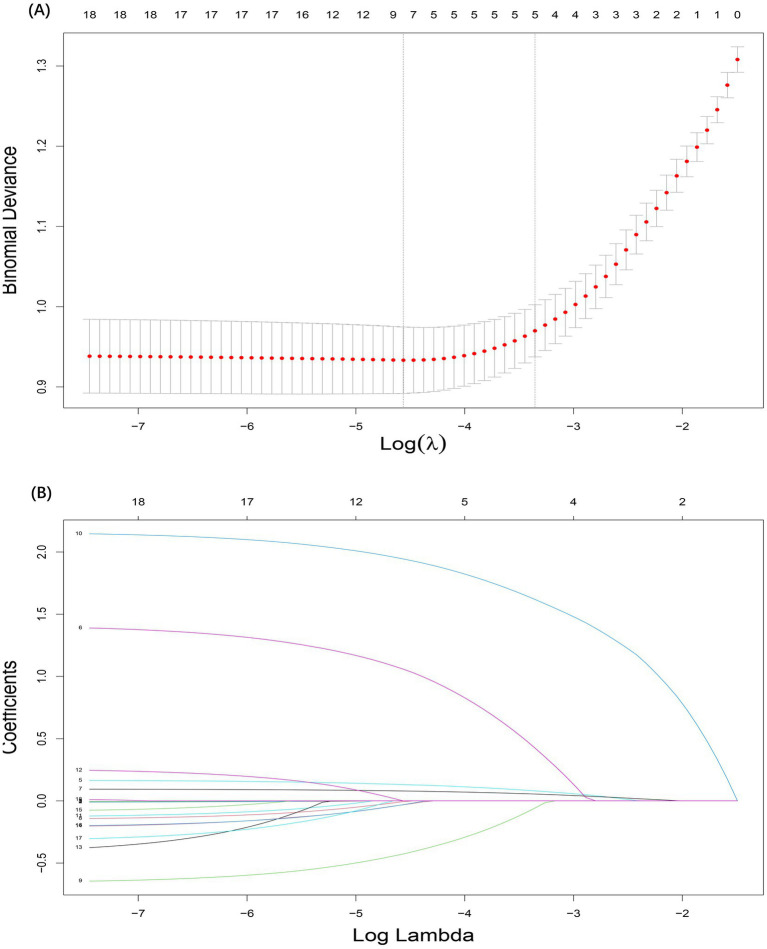
Selection process for candidate risk-factors using lasso regression. **(A)** The selection of the tuning parameter (lambda) in the lasso regression. **(B)** Lasso coefficient profiles of the 18 candidate predictors.

A total of 5 candidate predictors were selected using lasso regression analysis. The details of selection process, including lambda and coefficient profiles, for candidate risk-factors using lasso regression was shown in Figure 2. The β-coefficients of variables and the lambda min-value of lasso regression are detailed in Table 2. After multifactor logistic regression analysis, the results reported as odds ratio (95% CI), motion sickness (OR, 8.53; 95% CI, 6.21–11.81), anesthesia time (OR, 4.20; 95% CI, 2.09–8.65), fasting time (OR, 1.17; 95% CI, 1.13–1.22), anxiety score (OR, 1.10; 95% CI, 1.08–1.12) and artificial airway (OR, 0.54; 95% CI, 0.39–0.74) were independent predictors of PONV in patients undergoing day-case hysteroscopic surgery (Table 3).

**Table 3:** Results of multivariate logistic regression analysis of risk predictors for PONV.

Characteristics	β*-*coefficient	*SE*	*OR* (95% CI)	*P*
Motion sickness				
Yes	2.14	0.16	8.53 (6.23–11.81)	<0.001
No				
Anesthesia time (hr)	1.43	0.36	4.20 (2.09-8.65)	<0.001
Artificial airway				
Endotracheal intubation	−0.62	0.16	0.54 (0.39–0.74)	<0.001
Laryngeal mask				
Anxiety score (BAI)	0.09	0.01	1.10 (1.08–1.12)	<0.001
Fasting time (hr)	0.16	0.02	1.17 (1.13–1.22)	<0.001

PONV, postoperative nausea and vomiting; β-Coefficient, regression coefficients; SE, standard error; OR, odd ratios; CI, confidence intervals; BAI, beck anxiety inventory.

### Model construction

**Figure 3:**
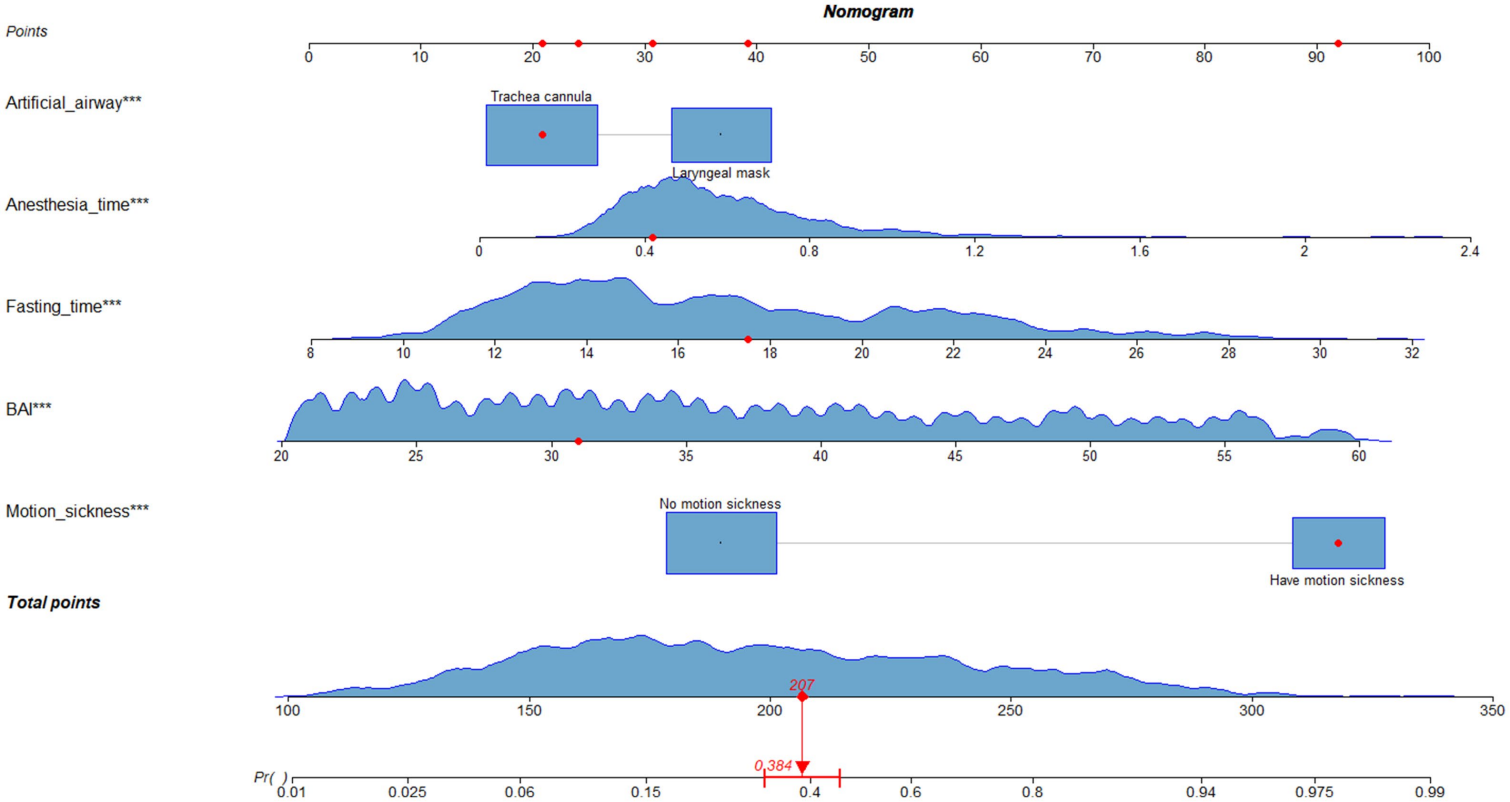
Static nomograms for risk prediction models.

**Figure 4:**
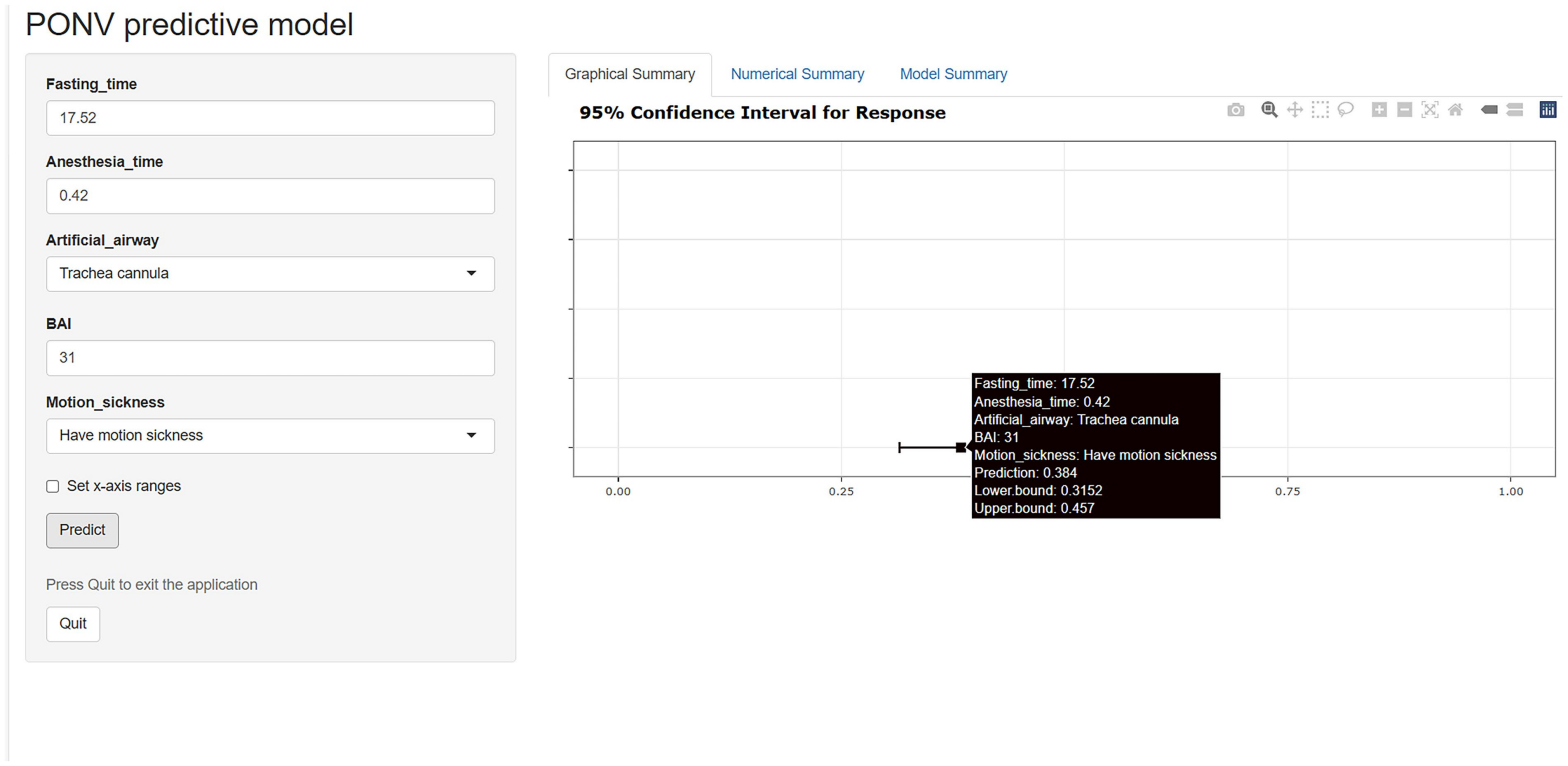
Screenshot of the web-based dynamic predictive model.

The nomogram of this predictive model is shown in Figure 3. The web-based dynamic nomogram is available online.^1^ The following patient is used to illustration. A patient with motion sickness, and endotracheal intubation was planned. Anxiety score was 31. Anesthesia time was 0.42 h. Fasting time was 17.52 h. The model predicted that the probability of PONV in patients was approximately 38.4% (Figure 4). The risk prediction model for PONV in patients undergoing gynecological hysteroscopic day-case surgery is as follows:

$P\;(\text{probability of PONV})=1/(1+e^{-Z}).$

*Z* = −8.29 + 2.14 (with motion sickness) + 1.43 (anesthesia time) + 0.16 (fasting time) + 0.09 (anxiety score) −0.62 (endotracheal intubation).

### Model validation

**Figure 5:**
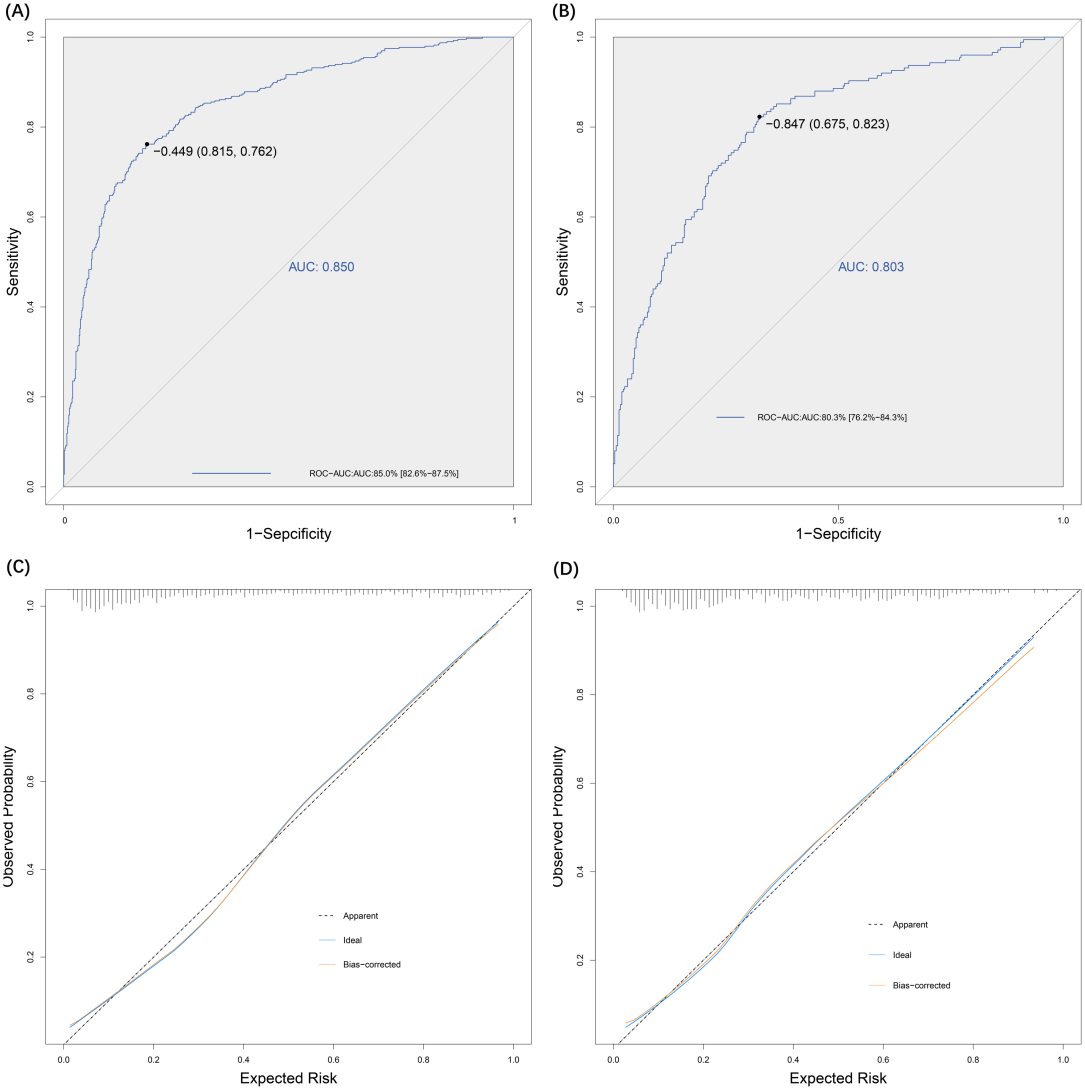
Effectiveness evaluation of risk prediction model. **(A)** ROC-AUC for the test group was 0.85. **(B)** ROC-AUC for the validation group was 0.80. **(C)** Evaluation of the goodness-of-fit of the model in the test group. **(D)** Evaluation of the goodness-of-fit of the model in the validation group.

**Figure 6:**
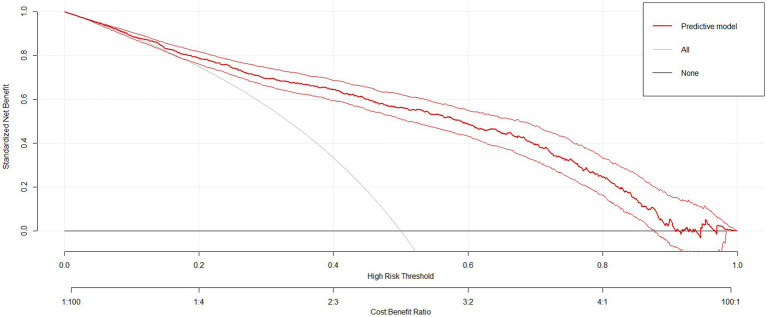
Decision Curve Analysis (DCA) for the risk prediction model.

The sensitivity and specificity of the risk prediction model were 0.76 and 0.82, respectively. The area under the ROC curve generated based on the training cohort was 85.0% (95% CI: 82.6–87.5%) and the validation cohort was 80.3% (76.2–84.3%), as shown in Figure 5. The calibration curves (Figure 5) and Hosmer-Lemeshow test (*X*^2^ = 12.568, *p* = 0.128) showed that the model has a good fit of goodness. DCA showed that the net benefit of this risk prediction model exceeded two extremes when the threshold probabilities were between 0.1 and 0.98 (Figure 6). Therefore, this risk prediction model can provide clinical benefits for patients undergoing day-case hysteroscopic surgery.

## Discussion

This is the specific-model to predict the risk of PONV in patients undergoing hysteroscopic day-case surgery. The model was helpful in the accurate identification of high PONV risk in patients undergoing hysteroscopic day-case surgery, and the early development of prevention and intervention programs. The developed predictive model was presented as a web-based nomogram, which is characterized by its simplicity and practicality compared to the usual nomogram or formulas. The incidence of PONV in all patients undergoing day-case hysteroscopic surgery was 36.0%. This also confirmed our previous conjecture that the risk of PONV in patients undergoing hysteroscopic day-case surgery is a non-negligible issue. The developed web-based dynamic predictive model incorporated five comprehensive and easily acquired predictors including motion sickness, anesthesia time, fasting time, anxiety, and artificial airway.

The correlation between motion sickness and PONV has been justified as a crucial influential predictor of PONV (1). Likewise, motion sickness was the primary factor for PONV in this study. Furthermore, we found that some patients just felt nausea in the PACU, but when they were transferred to the ward, their nausea would worsen and they even start vomiting. We considered that this might be partly related to patient movement. A study by pasini proved that 66% of PONV were due to the sudden change of position or movement (8). Additionally, anesthesia time was also a risk factor for PONV. It is notable that there were only 6.4% (101 out of 1,583) participants with anesthetized duration more than 1 h. This is because the anesthesia time for hysteroscopic surgery is very short. Moreover, there are studies reported that the increased occurrence of PONV might be partly attributed to pain and the use of N2O (17, 25). Some studies have found that PONV prevention should be considered in patients with anxiety sensitivity, and this finding was also obtained in our study. Patients with preoperative anxiety are more likely to suffer PONV (26), because severe anxiety may affect the sleep quality of patients and increase their sensitivity to PONV. Another predictor of PONV was the type of artificial airway. According to the findings of this study, endotracheal intubation could reduce the risk of PONV compared with laryngeal mask (β, −0.62; SE, 0.16; OR, 0.54; 95% CI, 0.39–0.74). This may be attributed to the incomplete airtightness of the laryngeal mask increasing the incidence of postoperative gastric distension. Tramer et al. explored a decision tree for PONV in females, in which the use of prophylactic antiemetics is necessary depending on the type of artificial airway (27, 28). Many studies have reported that prolonged fasting time can exacerbate the occurrence of PONV (29, 30), which was also confirmed in our study. Fasting time was one of the predictors for PONV in this study. The average fasting time of patients with PONV was longer than that of patients without PONV in our study. This may be attributed to the long fasting time increasing the risk of gastric acid irritation.

Among other risk factors not included in the prediction model, the effect of BMI on PONV has always been controversial. However, no significant association was found between BMI and PONV in our study. This may be due to the fact that all participants receiving hysteroscopy were female patients. While Choi DH et al. believe that it is gender rather than BMI itself that has a significant effect on PONV (31), which also explains the overweight/obesity does not lead to PONV (17, 32, 33). Some studies have reported that ASA classification is significantly associated with the occurrence of PONV, but this was not observed in our study. This may be due to the ASA classification of the participants in our study was generally 1 to 2 (32, 34). Previous studies have reported that the increasing age leads to a decline in autonomic reflexes, resulting in insensitive to PONV (35). However, this finding was not significant in the present study. Moreover, differences in methods of hysteroscopic surgery were not significant predictor of PONV in our study.

To ensure the reliability of the study results, the process of data collection and statistical analysis was carried out by professionally trained researchers who were blinded to the study’s purpose. More detailed variables were included in our study than in previous studies. Additionally, the sufficient sample size and more targeted study population contribute to providing more stable results. This may explain why our model demonstrates better discrimination. The intergroup differences of motion sickness in the baseline comparisons may be attributed to the observational study design and the use of time-period validation method to divide the modeling group and validation group. However, lasso regression analysis was used to eliminate the influence of confounders and interactions as much as possible in this study. General anesthesia is the most common form of anesthesia used today. However, we could not analyze the effect of different anesthesia methods on PONV, as all participants received general anesthesia. Smoking was not a predictor of PONV in our study. This may be attributed to the reluctance of the female study population to admit to smoking. Only 12 of the 1,583 patients admitted to smoking. Furthermore, non-smoking remains a controversial risk factor for PONV. Apfel et al. believed that smokers’ lower susceptibility to PONV was due to functional changes in neuroreceptors caused by nicotine withdrawal, rather than nicotine exposure (1, 36). Although the Apfel score is the most widely used model for predicting PONV (37), it may not be applicable to patients undergoing hysteroscopic day-case surgery. This is because the default existence of two (female and received Opioids during the surgery) of the four Apfel risk factors in patients undergoing hysteroscopic day-case surgery. This may decrease the effectiveness of Apfel risk forecasting for female patients receiving hysteroscopy. The ROC of this risk predictive model may not be ideal, which may be attributed to the influence of single-center study and potential confounders. Future studies are needed to further improve the risk prediction of PONV in day-case hysteroscopic surgery.

### Relevance to practice

The findings of this study are helpful for early screening of patients undergoing hysteroscopic day-case surgery at high risk of PONV by clinical providers. A web-based dynamic risk prediction model was constructed, with the advantage of simplicity and practicality, which contributes to the early development of prevention and intervention strategies.

### Limitation

The present study only considered risk factors from studies published up to 2024. Therefore, there may be other relevant predictors that were not analyzed. There may be limitations in the generalizability of our findings, as the participants were from a single PACU setting. Moreover, the study focused only on a specific subset of patients (those undergoing hysteroscopic day-case surgery), so the model may not be applicable to other types of surgeries or patient populations. Although the risk prediction model has been externally validated within the present research center, multi-center validation is needed for future studies. Since all participants received general anesthesia, the influence of other anesthetic methods or adjuncts could not be assessed. The occurrence of dependent variables needed to be collected with the help of telephone follow-up due to the nature of hysteroscopic ambulatory surgery. This may lead to some patients being lost to follow-up, which could affect the accuracy of the follow-up results.

## Conclusion

In summary, the web-based dynamic predictive model demonstrated potential in predicting the risk of PONV in patients undergoing day-case hysteroscopic surgery. However, external validation at other centers is required in future studies to assess its generalizability.

## Relevance to practice

This study emphasizes the importance of PONV risk prediction, potentially contributing to early risk prediction of PONV by clinical nurses in patients undergoing hysteroscopic day surgery.

## Patient or public contribution

Prior to the study, the investigator interviewed patients and obtained the written informed consent. Preoperatively, the motion sickness and anxiety of the patients were assessed and recorded by the investigators. Postoperatively, the occurrence of PONV in patients was documented by clinical observation and telephone follow-up. Other information was obtained from medical records.

## Data Availability

The datasets presented in this study can be found in online repositories. The names of the repository/repositories and accession number(s) can be found in the article/supplementary material.

## Footnotes

^1^ https://fsr0536.shinyapps.io/dynnomapp/

## References

[1] Apfel CC Heidrich FM Jukar-Rao S Jalota L Hornuss C Whelan RP. Evidence-based analysis of risk factors for postoperative nausea and vomiting. Br J Anaesth. (2012) 109:742–53. doi: 10.1093/bja/aes276, PMID: 23035051

[2] Gan TJ Diemunsch P Habib AS Kovac A Kranke P Meyer TA. Consensus guidelines for the management of postoperative nausea and vomiting. Anesth Analg. (2014) 118:85–113. doi: 10.1213/ANE.0000000000000002, PMID: 24356162

[3] Ugochukwu O Adaobi A Ewah R Obioma O. Postoperative nausea and vomiting in a gynecological and obstetrical population in south eastern Nigeria. Pan Afr Med J. (2010) 7:6. doi: 10.4314/pamj.v7i1.69111, PMID: 21954406 PMC3172643

[4] Canakci E Catak T Basar HE Cebeci Z Coskun I Saltali AO. Prevalence study for postoperative nausea vomiting: a training hospital example. Niger J Clin Pract. (2021) 24:1633–40. doi: 10.4103/njcp.njcp_399_20, PMID: 34782501

[5] Maraş G Bulut H. Prevalence of nausea-vomiting and coping strategies in patients undergoing outpatient surgery. J Perianesth Nurs. (2021) 36:487–91. doi: 10.1016/j.jopan.2020.10.004, PMID: 34167895

[6] Georgiou D Tranoulis A Jackson TL. Hysteroscopic tissue removal system (MyoSure) for the resection of polyps, sub-mucosal leiomyomas and retained products of conception in an out-patient setting: a single UK institution experience. Eur J Obstet Gynecol Reprod Biol. (2018) 231:147–51. doi: 10.1016/j.ejogrb.2018.10.030, PMID: 30388609

[7] Meulenbroeks D Hamerlynck TW Saglam-Kara S Van Rijssel NK Vliet HA Schoot BC. Hysteroscopic tissue removal systems: a randomized *in vitro* comparison. J Minim Invasive Gynecol. (2017) 24:159–64. doi: 10.1016/j.jmig.2016.08.829, PMID: 27597661

[8] Pasini A Belloni C. Intraoperative complications of 697 consecutive operative hysteroscopies. Minerva Ginecol. (2001) 53:13–20. 11279391

[9] Atieh AS Abu Shamma OK Abdelhafez MO Baniowda MA Abed S Babaa BH. Acute severe hyponatremia following hysteroscopic procedure in a young patient: a case report and review of the literature. Case Rep Nephrol. (2021):7195660. doi: 10.1155/2021/7195660 34594582 PMC8478601

[10] Istre O. Fluid balance during hysteroscopic surgery. Curr Opin Obstet Gynecol. (1997) 9:219–25. doi: 10.1097/00001703-199708000-00002, PMID: 9263711

[11] Odom-Forren J Jalota L Moser DK Lennie TA Hall LA Holtman J. Incidence and predictors of postdischarge nausea and vomiting in a 7-day population. J Clin Anesth. (2013) 25:551–9. doi: 10.1016/j.jclinane.2013.05.008, PMID: 23988801

[12] Elsaid RM Namrouti AS Samara AM Sadaqa W Zyoud S’H. Assessment of pain and postoperative nausea and vomiting and their association in the early postoperative period: an observational study from Palestine. BMC Surg. (2021) 21:177. doi: 10.1186/s12893-021-01172-9, PMID: 33794852 PMC8017875

[13] Pan PH Lee SC Harris LC. Antiemetic prophylaxis for postdischarge nausea and vomiting and impact on functional quality of living during recovery in patients with high emetic risks: a prospective, randomized, double-blind comparison of two prophylactic antiemetic regimens. Anesth Analg. (2008) 107:429–38. doi: 10.1213/ane.0b013e318172f992, PMID: 18633020

[14] Fetzer SJ Hand MA Bouchard PA Smith HB Jenkins MB. Self-care activities for postdischarge nausea and vomiting. J Perianesth Nurs. (2005) 20:249–54. doi: 10.1016/j.jopan.2005.05.001, PMID: 16102705

[15] Grabowska-Gaweł A Porzych K Piskunowicz G. Risk factors and frequency of postoperative nausea and vomiting in patients operated under general anesthesia. Przegl Lek. (2006) 63:72–6. 16969906

[16] Tatić M Skorić S Misković S Komarcević M Dobanovacki D Tomić G. Postoperative nausea and vomiting. Med Pregl. (2003) 56:431–5. doi: 10.2298/mpns0310431t 14740532

[17] Lerman J. Surgical and patient factors involved in postoperative nausea and vomiting. Br J Anaesth. (1992) 69:24s–32s. doi: 10.1093/bja/69.supplement_1.24S, PMID: 1486011

[18] Tan HS Cooter M George RB Habib AS. A risk score for postoperative nausea and/or vomiting in women undergoing cesarean delivery with intrathecal morphine. Int J Obstet Anesth. (2020) 44:126–30. doi: 10.1016/j.ijoa.2020.08.008, PMID: 32950029

[19] Amirshahi M Behnamfar N Badakhsh M Rafiemanesh H Keikhaie KR Sheyback M. Prevalence of postoperative nausea and vomiting: a systematic review and meta-analysis. Saudi J Anaesth. (2020) 14:48–56. doi: 10.4103/sja.SJA_401_19, PMID: 31998020 PMC6970369

[20] Dolin SJ Cashman JN Bland JM. Effectiveness of acute postoperative pain management: I. Evidence from published data. Br J Anaesth. (2002) 89:409–23. doi: 10.1093/bja/89.3.409, PMID: 12402719

[21] Apfel CC Läärä E Koivuranta M Greim CA Roewer N. A simplified risk score for predicting postoperative nausea and vomiting: conclusions from cross-validations between two centers. Anesthesiology. (1999) 91:693–700. doi: 10.1097/00000542-199909000-00022, PMID: 10485781

[22] Kappen TH Vergouwe Y Van Wolfswinkel L Kalkman CJ Moons KGM van Klei WA. Impact of adding therapeutic recommendations to risk assessments from a prediction model for postoperative nausea and vomiting. Br J Anaesth. (2015) 114:252–60. doi: 10.1093/bja/aeu321, PMID: 25274048

[23] Boogaerts JG Vanacker E Seidel L Albert A Bardiau FM. Assessment of postoperative nausea using a visual analogue scale. Acta Anaesthesiol Scand. (2000) 44:470–4. doi: 10.1034/j.1399-6576.2000.440420.x, PMID: 10757584

[24] Beck AT Epstein N Brown G Steer RA. An inventory for measuring clinical anxiety: psychometric properties. J Consult Clin Psychol. (1988) 56:893–7. doi: 10.1037/0022-006X.56.6.893, PMID: 3204199

[25] Kovac AL. Prevention and treatment of postoperative nausea and vomiting. Drugs. (2000) 59:213–43. doi: 10.2165/00003495-200059020-00005, PMID: 10730546

[26] Laufenberg-Feldmann R Müller M Ferner M Engelhard K Kappis B. Is 'anxiety sensitivity' predictive of postoperative nausea and vomiting?: a prospective observational study. Eur J Anaesthesiol. (2019) 36:369–74. doi: 10.1097/EJA.0000000000000979, PMID: 30865002

[27] Eghbal MH Sahmeddini MA. Comparison larygeal mask airway with the endotracheal tube for the external dacryocystorhionostomy surgery. A randomized clinical trial. Middle East J Anaesthesiol. (2013) 22:283–8. 24649784

[28] Tramèr MR. Strategies for postoperative nausea and vomiting. Best Pract Res Clin Anaesthesiol. (2004) 18:693–701. doi: 10.1016/j.bpa.2004.05.003, PMID: 15460553

[29] Hromatka BS Tung JY Kiefer AK do CB Hinds DA Eriksson N. Genetic variants associated with motion sickness point to roles for inner ear development, neurological processes and glucose homeostasis. Hum Mol Genet. (2015) 24:2700–8. doi: 10.1093/hmg/ddv028, PMID: 25628336 PMC4383869

[30] Yayla A Eskici İlgin V Kılınç T Karaman Özlü Z Ejder Apay S. Nausea and vomiting after laparoscopic cholecystectomy: analysis of predictive factors. J Perianesth Nurs. (2022) 37:834–41. doi: 10.1016/j.jopan.2022.01.002, PMID: 35382962

[31] Choi DH Ko JS Ahn HJ Kim JA. A korean predictive model for postoperative nausea and vomiting. J Korean Med Sci. (2005) 20:811–5. doi: 10.3346/jkms.2005.20.5.811, PMID: 16224155 PMC2779278

[32] Cohen MM Duncan PG Deboer DP Tweed WA. The postoperative interview: assessing risk factors for nausea and vomiting. Anesth Analg. (1994) 78:7–16. doi: 10.1213/00000539-199401000-00004, PMID: 8267183

[33] Apfel CC Roewer N. Risk assessment of postoperative nausea and vomiting. Int Anesthesiol Clin. (2003) 41:13–32. doi: 10.1097/00004311-200341040-00004, PMID: 14574212

[34] Sinclair DR Chung F Mezei G. Can postoperative nausea and vomiting be predicted? Anesthesiology. (1999) 91:109–18. doi: 10.1097/00000542-199907000-00018, PMID: 10422935

[35] Stephenson SJ Jiwanmall M Cherian NE kamakshi S Williams A. Reduction in post-operative nausea and vomiting (PONV) by preoperative risk stratification and adherence to a standardized anti emetic prophylaxis protocol in the day-care surgical population. J Family Med Prim Care. (2021) 10:865–70. doi: 10.4103/jfmpc.jfmpc_1692_20, PMID: 34041090 PMC8138419

[36] Latz B Mordhorst C Kerz T Schmidt A Schneider A Wisser G. Postoperative nausea and vomiting in patients after craniotomy: incidence and risk factors. J Neurosurg. (2011) 114:491–6. doi: 10.3171/2010.9.JNS10151, PMID: 21029035

[37] Balki M Carvalho JC. Intraoperative nausea and vomiting during cesarean section under regional anesthesia. Int J Obstet Anesth. (2005) 14:230–41. doi: 10.1016/j.ijoa.2004.12.004, PMID: 15935649

[38] Apfel CC Kranke P Eberhart LH. Comparison of surgical site and patient’s history with a simplified risk score for the prediction of postoperative nausea and vomiting. Anaesthesia. (2004) 59:1078–82. 15479315 10.1111/j.1365-2044.2004.03875.x

[39] Stamer UM Schmutz M Wen T Banz V Lippuner C Zhang L. A serotonin transporter polymorphism is associated with postoperative nausea and vomiting: An observational study in two different patient cohorts. Eur J Anaesthesiol. (2019) 36:566–74. 31274544 10.1097/EJA.0000000000001014

[40] Van den Bosch JE Moons KG Bonsel GJ Kalkman CJ. Does measurement of preoperative anxiety have added value for predicting postoperative nausea and vomiting? Anesth Analg. (2005) 100:1525–32. 15845719 10.1213/01.ANE.0000149325.20542.D4

